# Supplementary material for: Role of ciliopathy protein TMEM107 in eye development: insights from a mouse model and retinal organoid
Source: Life Sci Alliance. 2023 Oct 20;6(12):e202302073. doi: 10.26508/lsa.202302073 (PMC10589122; doi:10.26508/lsa.202302073)
Supplement: Supplementary file 1 [file LSA-2023-02073_TableS1.docx]

**SUPPLEMENTARY TABLE 1: Frequency in eye phenotype in *Tmem107^-/-^* embryos**

| **Stage** | **Number of *Tmem107^-/-^* embryos** | ***Anophthalmia*** | ***Microphthalmia*** | **Both eyes affected** |
| --- | --- | --- | --- | --- |
| E10.5 | 8 | 2 | 4 | 2 |
| E11.5 | 7 | 2 | 3 | 2 |
| E12.5 | 9 | 5 | 3 | 1 |
| E13.5 | 13 | 1 | 9 | 2 |
| E14.5 | 6 | 3 | 2 | 1 |
| E15.5 | 8 | 4 | 3 | 1 |
