# Supplementary material for: Role of ciliopathy protein TMEM107 in eye development: insights from a mouse model and retinal organoid
Source: Life Sci Alliance. 2023 Oct 20;6(12):e202302073. doi: 10.26508/lsa.202302073 (PMC10589122; doi:10.26508/lsa.202302073)
Supplement: Supplementary file 4 [file LSA-2023-02073_TableS2.docx]

**TABLE S2: Total number of analyzed embryos**

| **Stage** | **WT** | ***Tmem107^+/-^*** | ***Tmem107^-/-^*** |
| --- | --- | --- | --- |
| E10.5 | 4 | 7 | 8 |
| E11.5 | 8 | 8 | 7 |
| E12.5 | 6 | 5 | 9 |
| E13.5 | 6 | 18 | 13 |
| E14.5 | 9 | 6 | 6 |
| E15.5 | 4 | 10 | 8 |
