# Supplementary material for: Role of ciliopathy protein TMEM107 in eye development: insights from a mouse model and retinal organoid
Source: Life Sci Alliance. 2023 Oct 20;6(12):e202302073. doi: 10.26508/lsa.202302073 (PMC10589122; doi:10.26508/lsa.202302073)
Supplement: Supplementary file 5 [file LSA-2023-02073_TableS3.docx]

**TABLE S3: List of used primary antibodies.**

| **Antibody** | **Retrieval** | **Dilution** | **Cat. No.** | **Company** | **Notes** |
| --- | --- | --- | --- | --- | --- |
| PAX6 | Na-Citrate pH 6 | 1:50 | AB528427 | Developmental Studies Hybridoma Bank, United States | Mouse |
| SOX1 | Na-Citrate pH 6 | 1:100 | AF3369 | R&D Systems, United States | Mouse |
| SOX2 | Na-Citrate pH 6 | 1:200 | 2748S | Cell Signaling Technology, United States | Mouse |
| PAX2 | Na-Citrate pH 6 | 1:50 | NBP2-33496 | Novus Biologicals, United States | Mouse |
| ARL13B | Na-Citrate pH 6 | 1:100 | 17711-1-AP | ProteinTech | Mouse, Human organoids, ARPE-19 (1:400) |
| SHH | Na-Citrate pH 6 | 1:100 | ab73958 | Abcam, Cambridge, UK | Mouse |
| PAX6 | N/A | 1:200 | sc-53108 | Santa Cruz Biotechnology | Human organoids |
| RAX | N/A | 1:200 | sc-271889 | Santa Cruz Biotechnology | Human organoids |
| VSX2 | N/A | 1:200 | HPA003436 | Sigma-Aldrich | Human organoids |
| CRX | N/A | 1:200 | HPA036762 | Sigma-Aldrich | Human organoids |
| ARL13B | N/A | 1:200 | sc-515784 | Santa Cruz Biotechnology | ARPE-19 |
| GLI2 | N/A | 1:200 | 18989-1-AP | ProteinTech | ARPE-19 |
| SMO | N/A | 1:100 | sc-166685 | Santa Cruz Biotechnology | ARPE-19 |
| SOX2 | N/A | 1:200 | sc-365823 | Santa Cruz Biotechnology | Human organoids |
| PAX2 | N/A | 1:100 | NBP2-33496 | Novus Biologicals, United States | Human organoids |
| GLI3 | N/A | 1:500 | AF3690 | RD Systems, US | ARPE-19 |
| Vinculin | N/A | 1:1000 | 13901 | Cell Signaling Technology, US | ARPE-19 |
