# Supplementary material for: Role of ciliopathy protein TMEM107 in eye development: insights from a mouse model and retinal organoid
Source: Life Sci Alliance. 2023 Oct 20;6(12):e202302073. doi: 10.26508/lsa.202302073 (PMC10589122; doi:10.26508/lsa.202302073)
Supplement: Supplementary file 6 [file LSA-2023-02073_TableS4.docx]

**TABLE S4: List of primers for QPCR analyses**

| **Gene** | **Primer sequence** |
| --- | --- |
| ***GAPDH*** | FWD: TGCACCACCAACTGCTTAGC |
|  | REV: GGCATGGACTGTGGTCATGAG |
| ***RAX*** | FWD: GGCAAGGTCAACCTACCAGA |
|  | REV: CTTCATGGAGGACACTTCCAG |
| ***CRX*** | FWD: GTGAGGAGGTGGCTCTGAAG |
|  | REV: CTGCTGTTTCTGCTGCTGTC |
| ***PAX6*** | FWD: GCACACACACATTAACACACTTG |
|  | REV: GGTGTGTGAGAGCAATTCTCAG |
| ***SOX2*** | FWD: GGGGGAATGGACCTTGTATAG |
|  | REV: GCAAAGCTCCTACCGTACCA |
| ***VSX2*** | FWD: GGCGACACAGGACAATCTTTA |
|  | REV: TTCCGGCAGCTCCGTTTTC |
| ***MATH5*** | FWD: CCCTAAATTTGGGCAAGTGAAGA |
|  | REV: CAAAGCAACTCACGTGCAATC |
| ***RHODOPSIN*** | FWD: TTTGGAGGGCTTCTTTGCCA |
|  | REV: CCTCGGGGATGTACCTGGAC |
| ***RCVRN*** | FWD: TTCAAGGAGTACGTCATCGCC |
|  | REV: GATGGTCCCGTTACCGTCC |
| ***GLI1*** | FWD: GTGCACCACATCAACAGCGA |
|  | REV: GGTGCGTCTTCAGGTTTTCG |
| ***PTCH1*** | FWD: CTTCATGGCCGCGTTAATCC |
|  | REV: CTGACGCAGGGGCTTGTAAA |
| ***TMEM107*** | FWD: CGGGACAGCAACATACAGG |
|  | REV: CCTGCAGAGAGGAAGTGGAT |
| ***TMEM107 (FWD primer anneals to CRISPR/Cas9 targeted site)*** | FWD: ATGGGCCGGGTCTCAGG |
|  | REV: CCTGCTTGTCATACTCCTCGG |
